# Editors’ Book Table

**Published:** 1872-05

**Authors:** 


### Editors' Book Table.

[NOTE. — All works reviewed in the columns of the CHICAGO MEDICAL JOURNAL may be found in the extensive stock of W. B. KEEN, COOKE & CO., whose catalogue of Medical Books will be sent to any address upon request.]

BOOKS RECEIVED.

*A Treatise on the Diseases of Infancy and Childhood.* Second edition, enlarged and thoroughly revised. By J. LEWIS SMITH, Curator to the Nursery and Child's Hospital, New York, Clinical Lecturer on Diseases of Children, Professor in Bellevue Hospital Medical College, etc., etc. Philadelphia: Henry C. Lea. 1872. Pp. 741.

The high favor with which the first edition was received, renders unnecessary any formal introduction of this treatise to our readers. The present edition has been enlarged something over a hundred pages, nearly twenty additional diseases being treated of, and many new formulæ included. Other portions, especially those relating to pathological histology, have been re-written to correspond with recent discoveries. Undue augmentation of the bulk of the volume has been avoided by condensation of portions of the text of a less practical character. As it is now presented, the book is one we can sincerely recommend as both creditable to the author and more than usually profitable to the studious practitioner.

*A Treatise on Diseases of the Bones.* By THOMAS M. MARKOE, M.D., Prof. of Surgery in the College of Physicians and Surgeons, Surgeon of the New York Hospital, Surgeon of Bellevue Hospital, etc., etc. New York: D. Appleton & Co. 1872. Pp. 416.

In his very modest preface, Prof. Markoe informs us that his book contains the substance of the lectures which, for the last twelve years, he has delivered at the College of Physicians and Surgeons in New York. It is not claimed as a full and systematic exposition of the subject, but rather offers the fruits of his own experience and studies. Those who have the advantage of the author's personal acquaintance, will know at once the conspicuous value of his work. To those who do not, we confidently recommend it as a clear, concise, and, indeed, excellent exposition of the subject. Its free use of illustrations, together with Prof. Markoe's graphic descriptions, will help to clear away the obscurity which hangs around many points in this important department.

Read, mark, learn, and inwardly digest.

*The Urine and its Derangements, with the Application of Physiological Chemistry to the Diagnosis and Treatment of Constitutional as well as Local Disease.* By GEORGE HARLEY, M.D., F.R.S., late Professor in the University College, London, etc. With illustrations. One volume. Price, $2.75. Philadelphia: Lindsay & Blakiston. 1872.

Chapter 1. What is Urine? 2. Changes in the Composition of the Urine, induced by Food, Drink, Medicine, and Disease. 3. Urea, Ammonæmia, uræmia. 4. Uric Acid. 5. Hippuric Acid, Chloride of Sodium. 6. Urohæmatin, Abnormal Pigments in Urine. 7. Phosphoric Acid, Phosphatic Gravel and Calculi. 8. Oxalic Acid, Oxaluria, Mulberry Calculi. 9. Inosite in Urine, Creatin and Creatinine, Cholesterin, Cystin, Xanthin, Leucin, Tyrosin. 10. Diabetes Melitus. 11. Albuminuria.

The subject-matter of this volume was delivered in a course of lectures before the class at the University College, London, and published in detached portions in the *London Medical Times and Gazette*, where they were so favorably received that the author has been induced to revise and enlarge them, presenting them in a far more accessible form to the profession. Professor Harley's book now offers facilities for the study of Physiological and Pathological Chemistry, as applied to a class of diseases that is otherwise very imperfectly provided for.

*Clinical Lectures on the Diseases of Women.* By SIR JAMES Y. SIMPSON, Bart., M.D., D.C.L., Late Professor of Midwifery in the University of Edinburg. Edited by ALEX'R R. SIMPSON, M.D., Prof. of Medicine and Midwifery, and the Diseases of Women and Children in the University of Edinburg. New York: D. Appleton & Co., 549 and 551 Broadway. 1872. Pp. 789.

This is a continuation of the series of volumes containing the works of the eminent author afforded by the American publishers in elegant dress for American readers. It opens with a lecture on the Diagnosis of Diseases of Women, the balance being made up of Clinical Lectures. A copious index is subjoined. It is needless to commend the works of Sir. James Y. Simpson to the intelligent professional reader.

PAMPHLETS.

*Table I. Mortality of the United States (by States and Territories), with Distinction of Sex and Percentage of Deaths to Population, at the Censuses of* 1870, 1860 *and* 1850.

We acknowledge, with thanks, advance sheets of these tables, which are to form a part of the volume on the Vital Statistics of the United States, Ninth Census, 1870.

The information afforded cannot fail to prove of immense practical value to all earnest students of the profession, as well as to statesmen and philanthropists.

*Proceedings of the Homœopathic Medical Society of Ohio.* Seventh Annual Session at Cincinnati, 1871.

*Bulletin No.* 21. *April*, 1872. From the officers and trustees of the Boston Public Library.

*The Lens.* A Quarterly Journal of Microscopy and the Allied Natural Sciences; with the Transactions of the State Microscopical Society of Illinois. Edited by S. A. Briggs. Publishing Committee: Charles Briggs, E. H. Sargent, Charles Adams. Chicago, April, 1872. Vol. 1. No. 2. $3.00 per annum, in advance.

